# Current and future developments in the treatment of virus-induced hypercytokinemia

**DOI:** 10.4155/fmc-2016-0181

**Published:** 2017-02

**Authors:** Jonathan P Wong, Satya Viswanathan, Ming Wang, Lun-Quan Sun, Graeme C Clark, Riccardo V D'Elia

**Affiliations:** 1 Defence Research & Development Canada, Suffield Research Centre, Ralston, Alberta, Canada; 2 College of Veterinary Medicine, China Agriculture University, Beijing, China; 3 Center for Molecular Medicine, Xiangya Hospital, Central South University, Changsa, China; 4 CBR Division, Defence Science & Technology Laboratory, Porton Down, Salisbury, UK

**Keywords:** emerging viruses, hypercytokinemia, treatment

## Abstract

Emerging pathogenic viruses such as Ebola and Middle Eastern Respiratory Syndrome coronavirus (MERS-CoV) can cause acute infections through the evasion of the host's antiviral immune responses and by inducing the upregulation of inflammatory cytokines. This immune dysregulation, termed a cytokine storm or hypercytokinemia, is potentially fatal and is a significant underlying factor in increased mortality of infected patients. The prevalence of global outbreaks in recent years has offered opportunities to study the progression of various viral infections and have provided an improved understanding of hypercytokinemia associated with these diseases. However, despite this increased knowledge and the study of the infections caused by a range of emerging viruses, the therapeutic options still remain limited. This review aims to explore alternative experimental strategies for treating hypercytokinemia induced by the Ebola, avian influenza and Dengue viruses; outlining their modes of action, summarizing their preclinical assessments and potential clinical applications.

**Figure 1.**
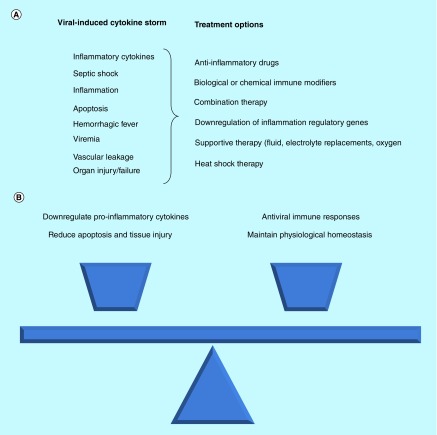
**Physiological challenges associated with developing an effective treatment for viral-induced cytokine storm.** **(A)** Summary of potential treatment options against virus-induced cytokine storm. **(B)** Importance of maintaining delicate balance between implementing anti-inflammatory measures and maintaining functional antiviral immune responses.

Emerging and re-emerging viruses have caused numerous global public health crises in recent years, and continue to threaten public health and security. Outbreaks associated with the person-to-person or animal-to-person transmission of Ebola, Middle Eastern Respiratory Syndrome coronavirus (MERS-CoV), Severe Acute Respiratory Syndrome (SARS) and Pandemic Influenza represent a few examples of this growing problem. These viruses are often difficult to protect against due to the lack of approved antiviral drugs and vaccines and the general population has limited herd or acquired immunity to provide protection from an infection. Additionally, they cause acute infections with high case fatality rates that are dependent upon the specific strain of virus but typically 50–90% for Ebola [1], 40% for MERS-CoV [2] and 60% for the HPAI [3]).

One of the underlying factors which may contribute to the high fatality rate is the ability of these viruses to induce hypercytokinemia, often termed a cytokine storm (CS), in immunocompetent individuals [3–5]. Cytokine storm is a potentially fatal immune condition characterized by rapidly proliferating and highly activated T cells, macrophages and natural killer cells and an associated overproduction and release of more than 150 inflammatory cytokines, and chemical mediators by immune or nonimmune defense cells [6,7]. If left untreated CS can lead to severe pathological complications including sepsis, shock, tissue damage and/or multiple organ failure, edema and ultimately death. Furthermore, CS has known to be closely associated with the development of viral hemorrhagic fevers which are a severe immunopathological condition caused by a number of viruses; including Ebola, Marburg, Lassa, Crimean-Congo hemorrhagic fever and Dengue fever (DF) viruses [8]. Despite the severity and potential fatal outcome following infection with these viruses, CS has seldom been specifically targeted in order to either diagnose and/or potentially treat the diseases that they cause. As there are generally no licensed antiviral drugs against most of these emerging viruses the treatment of these patients has been generally limited to supportive care such as fluid and electrolyte replacements. In developing countries this basic supportive care may also be absent.

Although the clinical presentations of CS symptoms can be markedly similar, treating virus-induced CS the same regardless of etiological agent agent may be challenging as a consequence of differences in virus type, exposure route, site of infection, pathogenesis and in the cytokine fingerprint each virus induced within the host. However, given these challenges, CS still represents one of the few truly broad-spectrum targets and therefore the treatment options in this review will be discussed in the context of each respective viral disease. As CS treatment is still in its early discovery phase, a better understanding of the immunological, biochemical and physiological interplay and the in the CS triggered by these viruses is expected to result in more effective and safer treatment options for patients.

Global outbreaks of emerging viral diseases in recent years had led to an improved understanding of the CS by specialists in infectious diseases along with a recognition of the importance of the condition by healthcare providers. In some rare cases the treatment of CS has been attempted in a small number of patients. However, these few exploratory studies yielded inconclusive results with varying therapeutic effects and outcomes [9,10]. This highlights the potential benefits and challenges of targeting the immune response for the treatment of viral infections. Using strategies that have been adopted for three specific emerging or re-emerging viruses, namely Ebola, avian influenza and Dengue, this review will outlines several treatment options that have been utilized that target CS (summarized in Table 1). The use of these therapeutic strategies within both experimental models of infection within laboratory animals and that have been undertaken in humans in a clinical setting are discussed and highlighted in this review.

## Pandemic influenza

Highly virulent influenza viruses are known to cause aberrant over-responsive immune responses which are linked to uncontrolled overexpression of pro-inflammatory cytokines/chemokines (acute hypercytokinemia) [6,7,11]. Influenza virus-induced CS is generally manifested by hyperinduction of inflammatory and apoptotic cytokines, infiltration and activation of T-effector cells with fluid accumulation in the lungs. The net effect of these immunological and physiological changes can result in airway blockage through acute respiratory distress syndrome (ARDS), multiple organ failures and/or shock which can potentially lead to death.

One of the earliest viral disease outbreaks to attribute CS as a cause of mortality associated with infection was the 1917–1918 Spanish influenza pandemic [12–14]. This pandemic is considered to be the most fatal in human history resulting in the loss of 50–100 million human lives worldwide [14]. Analysis of the age-related death rates for this pandemic revealed that infected people 15–34 years of age (teenagers and young adults) were more than 20-times higher than in previous influenza outbreaks. Overall, half of the influenza-related deaths were in young adults 20–40 years of age [14]. This is in stark contrast to the mortality versus age curve observed for seasonal influenza strains, which preferentially cause deaths in the elderly and persons with underlying medical conditions (e.g. immunocompromised).

It is noteworthy that a similar trend in the disproportionate high incidence and death rates in the under 35 years age group were observed during the highly pathogenic avian H5N1 influenza A (HPAI) virus outbreaks in East Asia from 2004 to 2007. The HPAI virus was primarily found in domesticated poultry and wild migratory birds, the natural host reservoir for the virus, spreading to humans who came into close contact with infected birds. With a case fatality rate of 50–60%, HPAI is one of the most virulent influenza viruses surpassing the fatality rate of the 1917–1918 pandemic influenza [3]. Despite its high fatality rate, the HPAI virus lacks the ability for direct human-to-human transmission limiting its natural spread as well as the numbers of lives lost during the outbreak [15].

Although oseltamivir had been given to several patients infected with HPAI during the HPAI outbreaks in Southeast Asia, no definitive conclusions about the therapeutic efficacy can be derived from these studies [3]. However, this clinical evaluation did not suggest a significant decrease in mortality by antiviral treatment with oseltamivir [3,11]. Cumulative reported studies suggested the survival rate in treated patients was 30% (13 of 34) compared with 25% (4 of 16) in untreated patients. The lack of a significant increase in survival within treated patients may be attributable to the timing of administration of the antiviral treatment. Patients were only treated during the later stages of the infection when the presence of irreversible pulmonary damage/failure and/or pathological changes associated with the immune hyperinduction was likely to be present. Indeed the efficacy of oseltamivir treatment was dependent on how early the drug treatment was administered; survival rates of 53% were found for patients treated within 5 days of onset of illness and decreased to 26% administration commenced after 6 days [3,11].

## Broad acting anti-inflammatory drugs as treatments for the CS induced by pandemic influenza viruses

In order to successfully treat infections caused by viruses such as pandemic influenza combination strategies are required in order to reduce mortality in patients by both directly, inhibiting viral spread or replication (i.e. through the administration of antivirals) and that also mitigate the effects of CS (i.e. anti-inflammatory drugs) without impeding the natural ability to fight infection. Collectively, this approach represents a promising approach for treating viral infections.

A number of anti-inflammatory drugs (administered as either as mono- or combination therapies) have been evaluated for the treatment of CS during infections with influenza. However, one example illustrates the therapeutic challenges associated with administering anti-inflammatory drugs as monotherapies for treating acute viral infections. It was found that glucocorticoid treatment administered for the suppression of cytokine production did not protect mice from lethal infection with HPIA (A/Vietnam/1203/04 H5N1) [16]. Further, in human case studies during the H5N1 outbreaks in Southeast Asia patients who were treated with steroids such as dexamethasone and methylprednisolone (with or without the antiviral drug oseltamivir) were found to have high mortality rates and steroid treatment did not improve clinical outcomes [9]. The use of COX-2 inhibitors (celecoxib, mesalazine), both as a monotherapy and in combination with the anti-influenza drug (neuraminidase inhibitor zanamivir), has also been considered within experimental models of influenza. It was found that COX-2 inhibitors enhanced the antiviral efficacy of zanamivir in the treatment of mice against infection with influenza A/H5N1 virus [17]. In this study, all treatments were administered at 48 h post challenge to mimic the timely lines associated with diagnosis in a clinical setting. Under this treatment regimen, the use of zanamivir alone did not result in significant improvement in the survival rate (13.3%), but enhanced survival (10.7 days) when compared with the untreated control group (0% survival at 6.6 days) [17]. Zanamivir alone did reduce virus load in the lungs, but not inflammation and mortality. Similarly, the use of COX-2 inhibitors alone (celecoxib or mesalazine, or both) did not provide any improvement in the survival rate (0% survival) but prolonged survival time (between 8.5 and 9.5 days compared with 6.6 days for control). However, when the infected mice were treated with a triple combination of zanamivir, celecoxib and mesalazine administered at 4 h post virus challenge, there were significant improvements in the survival rate (53.3%), prolongation of the survival time (13.3 days), compared with the zanamivir alone administered at 4 h post-virus challenge (13.3% survival and 8.4 days survival time). Additionally, it was found that the treatment of mice with the triple drug combination also resulted in higher levels of CD4^+^ and CD8^+^ T lymphocytes and reduced pulmonary inflammation [17]. The results from this study suggest that the combination treatment of zanamivir with COX-2 inhibitors can significantly improve survival outcome and that this combination therapy can provide synergistic therapeutic outcomes by reducing cytokine dysfunction and preventing apoptosis [17,18].

Recently, the experimental use of sphingosine-1-phosphate-1 receptor (S1P1R) agonists for the suppression of CS and as a treatment for pathogenic influenza virus infection in mice has been described [19,20]. SIP1R agonists (specifically CYM5442 and AAL-R) are novel therapies that have been administered to mice following infection with human pandemic influenza A/Wisconsin/WSLH34939/09 and found to inhibit the cellular and cytokine/chemokine responses to limit the immunopathogenic damage, as well as providing significant protection (82% survival, 23/28 mice) against an otherwise lethal virus challenge [19]. This level of protection was higher when compared with treatment with the antiviral drug oseltamivir (50% survival, 14/28 mice), although a combination therapy approach involving the administation of both AAL-R and oseltamivir provided the highest level of protection (96% survival rate, 27/28 mice) [19].

Mechanistic studies in mice have demonstrated that the efficacy of S1P1R agonist in suppressing CS occurs as a consequence of the systemic inhibition downstream of the myeloid differentiation primary response gene (MyD88) and IFN-β promoter stimulator-1 signaling [21]. MyD88 a key component of the innate immune system was identified in the study as the adaptor molecule of the TLR signaling pathway responsible primarily for the cytokine amplification following pathogenic influenza virus challenge. S1P1R agonist AAL-R has shown to significantly reduce the release of inflammatory cytokines while maintaining protective antibody and cytotoxic T-cell responses in mice [21]. These preclinical data highlight the promise of these drugs for treating acute viral infections and suggest that S1P1R agonists could improve clinical outcomes and significantly reduce mortality of patients infected with the highly pathogenic or pandemic influenza by controlling the fatal CS side effects of disease.

S1P1R agonists will now be required to undergo future development leading to safety and dose tolerability testing in Phase I clinical trials before consideration can be given to their use in humans. However, if these potential drug products are found to have good safety profiles there will be a compelling justification for these candidate drugs to be taken forward for full regulatory approval. If these drugs should reach licensure it would greatly enhance our pandemic preparedness not only against influenza but also potential for combating other emerging viral diseases that induce CS (e.g., Ebola, MERS-CoV and SARS).

## Targeted silencing of regulatory gene *c-Jun* with DNAzymes to modulate inflammation during influenza infection

An innovative approach to treat CS may encompass downregulation of key control genes/signaling pathways that regulate the inflammatory and/or apoptosis responses in the host. The c-Jun N-terminal Kinases signaling pathway is involved in the regulation of numerous cell activities including proliferation, apoptosis and inflammation, and is reported to be activated during influenza A virus infection and replication [22]. c-Jun is a downstream molecule of c-Jun N-terminal Kinases and the increased expression of the *c-Jun* gene has been observed in proliferation, apoptosis and tumorigenesis. During infection with influenza H5N1 virus the c-Jun protein is activated to its phosphorylated form and enhances the transcription of cytokines such as TNF-α and IFN-β and the chemokine RANTES which contributes to cytokine overproduction; collectively resulting an increase in inflammation [23].

DNAzyme are synthetic, single-stranded DNA that are designed to catalyze the degradation of mRNA through complimentary binding via Watson-Crick base pairing [24]. DNAzyme cleaves the mRNA at predetermined phosphodiester linkages [24]. DNAzyme 13 (DZ 13) is a catalytic DNA that specifically cleaves *c-Jun* mRNA [25]. DZ 13 has been shown in a number of preclinical studies to exhibit anti-inflammatory activity in animal sepsis [26] and tumor growth [26] models. DZ 13 had also been shown to be safe and well tolerated in Phase I clinical testing in normal human volunteers [27].

In a pilot study using a murine infection model with the highly pathogenic avian influenza H5N1 A virus, it was shown that DZ 13 inhibited c-Jun activation and led to improved protection and survival rate of mice against the infection [23]. Additionally, the DZ 13 suppression of c-Jun also decreased CD8^+^ T-cell proliferation, pro-inflammatory cytokine expressions, reduced virus titer and tissue histopathology such as inflammatory cell infiltration and interstitial edema in the lung [23]. Taken together, the promising results show that suppression of key inflammatory gene expression, such as DZ 13 knockdown of *c-Jun* gene expression, can potentially be a powerful strategy for the downregulation of inflammation responses (CS) and leads to enhanced protection and therapeutic outcomes against severe and highly fatal virus infection including the HPIA virus [23]. Furthermore, it is postulated that DZ 13 may have broad-spectrum anti-inflammatory effects. It is also possible to explore whether this approach of downregulating the cytokine overproduction can have synergistic therapeutic effect when used in combination with an antiviral drug such as oseltamivir or zanamivir. However, further animal studies are warranted to support these concepts.

## Protective role of heat shock treatment for hypercytokinemia in HPAI infection

Short-term heat shock, as a stress inducer, has been known to induce the expression of heat shock proteins (HSPs) that function as molecular chaperones to protect cells from multiple stresses. HSPs also play an important role in regulating innate and adaptive immune responses, including activating monocytes, dendritic cells, macrophages and inducing production of pro-inflammatory cytokines (review, [28]). Short-term heat shock (39°C for 4 h) treatment of mice resulted in significant increased levels of HSP70 (a key member of the HSP protein family known to play in a role in antiviral immunity), and in the levels of pro-inflammatory cytokines IL-6, TNF-α, IFN-β and IFN-γ [29]. However, when the heat shock-treated mice were challenged with a lethal dose of HPAI virus, 57% of the heat shock-treated mice survived the virus challenge at day 14 post-infection compared with 0% survival in the untreated control group [29]. The heat shock-treated mice were found to have reduced lung pathological damages, reduced body weight loss and virus replication in the lung tissues which was found to correlate with an increased expression of HSP70 in the lungs of the heat shock-treated group when compared with the control [29]. Intriguingly, the expression of pro-inflammatory cytokines IL-6, IFN-β and IFN-α were significantly reduced at day 6 and a reduction in the presence of TNF-α level was also noted at day 1 post-infection within the lungs of mice which received the heat shock treatment when compared with the untreated control group [29]. The beneficial effects of heat shock treatment against HPAI in this study was hypothesized to be correlated with the increased expression and protective effects of HSP70 which was also associated with transient downregulation of the over-expressions of pro-inflammatory cytokines in the lungs.

### Antiviral treatment of Ebola

Ebola virus causes a highly lethal hemorrhagic fever, and is among the most deadly human pathogens. The most virulent of Ebola virus, Zaire strain, has a case fatality rate of up to 90% [30]. The highly fatal outcome of infection with Ebola Zaire strain in humans had been associated with aberrant innate responses, characterized by CS, with hypersecretion of numerous pro-inflammatory cytokines, chemokines and growth factors [5]. The physiological effects induced by CS are thought to lead to disseminated intravascular coagulation, vascular dysfunction and collapse, along with multiple organ failure and shock-like syndrome associated with fatal outcome [5].

The Ebola outbreaks in the African continent from 2014 to the present have so far infected more than 28,000 people with more than 11,000 fatalities [31]. Despite the devastating toll of Ebola on human lives there are still no approved antiviral drugs for the treatment of human Ebola. Several experimental drugs were evaluated in human patients on an emergency compassionate basis and these include ZMapp (monoclonal antibody cocktails), TKM-Ebola (small interference RNA [siRNA] cocktails) and favipiravir (anti-influenza drug) (review, [32]). However, to date, there has been no definitive report of a highly effective therapy for Ebola in humans. This is partly due to the fact that there were no standard randomized controlled trials undertaken as most drugs were administered on a compassionate basis. Each of these experimental drugs used specifically targets the Ebola virus and are not designed to treat immunological dysfunction of cytokine overexpression.

### Supportive therapies of Ebola patients

Despite the uncertainty of the efficacy of experimental antiviral therapy, the Ebola crisis had revealed the beneficial role of supportive therapy including fluid and electrolyte replacement, as well as oxygen therapy [33]. In one such study in Guinea in 2014, oral rehydration was given to 36 patients with confirmed Ebola, and 28 patients of these patients received additional intravenous fluid resuscitation. A median of 1 l of intravenous crystalloids was administered to these patients during the first 24 h after admission. Of these patients who received supportive therapy, the mortality rate was 43% (16 of 36 patients) [33]. This case fatality rate here is lower than that recorded at other sites in Guinea [33]. Another retrospective study of 106 Ebola patients from Sierra Leone, documented a case fatality rate of 74%. High-quality supportive care is thought to have contributed to the higher number of survivors observed in Guinea. More randomized and controlled studies are needed to affirm the therapeutic role of supportive therapies such a fluid and electrolyte replacements in improving survival in the treatment of Ebola patients.

It is thought that general supportive care and therapy including fluid rehydration and electrolyte replacement may mitigate hemorrhage, septic shock and reduce multiple organ failure. These clinical manifestations are associated with the detrimental effects of cytokine and immunological dysfunctions, it follows that the supportive therapy may play a beneficial role in reducing the clinical damages induced by CS.

### Experimental treatment of CS in Ebola infection

A number of studies involving a variety of experimental drugs that suppress cytokines have been undertaken in animal/cell culture infection models of Ebola, including myxomavirus-derived serpin [34] and pyridinyl imidazole inhibitors of p38 MAPK [35]. Myxomavirus is an enveloped DNA virus belonging to the poxviridae family. It causes fatal myxomatosis in rabbits by evading the host's immune response, and causing pathological damages. The virus produces a number of extracellular proteins which protects the virus from destruction by the host's antiviral immune responses. One of these proteins, SERP-1, is a potent inhibitor of both thrombolytic and thrombotic proteases and has been demonstrated to have anti-inflammatory properties in a wide range of animal models [36,37]. In a small exploratory study, wild-type BALB/c mice infected with mouse-adapted Ebola virus (Zaire 1976 Mayinga strain) treated with SERP-1 was found to increase survival time/rate and this was dose dependent, with higher drug concentration offering higher protection [34]. Furthermore, treated animals were found to have decreased tissue necrosis and cell invasion in both spleen and livers, compared with untreated infected mice [34]. In addition, it is noteworthy that purified SERP-1 protein has been demonstrated to have anti-inflammatory activity in animal models of vascular injury highlighting its potential broader therapeutic utility.

These preliminary findings may provide important insights on whether reducing inflammatory and tissue damage mediated by Ebola virus can result in improved therapeutic outcomes, and supports a larger scale preclinical study to determine whether SERP-1 can be used to treat Ebola virus-induced deaths in larger animal models including guinea pig and non-human primates.

Ebola virus targets antigen presenting cells including macrophages and dendritic cells, and in the process, also activates the p38 and MAPK signaling pathways [35]. The p38 and MAPK signaling pathways play an essential role in immune evasion and induction of the deregulated immune response, and present attractive targets for downregulating the inflammatory and enhance antiviral response to infectious viruses. When Ebola virus targets and infects these antigen-presenting cells, they can effectively knock down type 1 interferon production, but upregulate the production of a number of pro-inflammatory cytokines including TNF-α, IL-1, IL-6, IL-8, IL-15 and others). These combined effects result in the impairment of the innate immune responses and enhance viral replication in the host.

A number of p38 MAPK chemical inhibitors have been identified and these have been tested in monocyte-derived dendritic cells for their ability to inhibit Ebola virus infection and/or cytokine overproduction [35]. Indeed, pyridinyl imidazole inhibitors were found to reduce Ebola virus replication in PMA-differentiated human THP-1 cells and/or in a primary monocyte-derived dendritic cells [35]. Furthermore, the production of a number of cytokines, including TNF-α, IL-6 and IL-15, was found to be inhibited in a dose-dependent manner. Furthermore, a number of chemokines associated with fatal outcomes in humans, for example, MIP-1α, MIP-1β and RANTES were also found to be reduced [35].

Furthermore, a recent publication has suggested that repurposing of the generic, nonspecific anti-inflammatory therapy ibuprofen could also be used for prevention and treatment of Ebola [38]. However, it is proposed that ibuprofen may work by destabilizing glycoprotein of the virus thereby preventing virus attachment and cellular entry [38]. Additional studies are warranted to determine whether this potential dual action of ibuprofen glycoprotein destabilization and as an established anti-inflammatory demonstrate significant therapeutic benefit within experimental animal models of infection and/or within humans cases of Ebola.

### Antibiotic treatment and their effects on CS in Dengue hemorrhagic fever

Infection by the mosquito-borne Dengue virus can progress from an asymptomatic phase termed DF to the more severe forms of Dengue hemorrhagic fever (DHF) and shock syndrome. If left untreated these inflammatory-driven responses can to sepsis and death. When first infected by the four serotypes of Dengue virus (DEV-I to DEV-V) the majority of cases tend to be asymptomatic or mild. Complications can arise when the individual is subsequently re-infected with a different serotype of DEV, where the disease becomes more severe and can lead to potentially fatal DHF and Dengue shock syndrome. In patients with severe forms of Dengue the serum levels of several cytokines including IL-1, IL-4, IL-6, IL-7, TNF-α, IFN-γ and IL-13 are elevated and the severity of disease is thought to be direrctly linked to increases in the inflammatory cytokine [39]. The aberrant cytokine levels are known to be associated with increased incidence in the development of DHF and thus suppressing cytokine levels has been used as an approach to improve therapeutic outcome in Dengue patients.

In a small pilot study, doxycycline and tetracycline has been evaluated for their effectiveness to modulate inflammatory cytokines in patients with DHF or DF [40]. In a randomized study, serum levels of various cytokines in doxycycline- or tetracycline-treated Dengue patients were compared with that of the untreated control patients. Patients receiving doxycycline or tetracycline treatment were demonstrated to have significant reductions in their serum levels of IL-1, IL-6 and TNF-α, compared with untreated control patients. Downregulation of these cytokines was observed to be persistent and continued for between 3 days to 7 days following treatment [40]. Furthermore, doxycycline was found to be more effective than tetracycline in the modulation of these pro-inflammatory cytokines and resulted in the greater reduction in serum cytokine levels [40].

The mechanism by which doxycycline modulates inflammatory cytokines in patients infected with DF virus is not completely understood. Doxycycline is an antibiotic belonging to the class of tetracyclines which are known to possess known immunomodulating effects, including reducing serum levels of these pro-inflammatory cytokines in patients with multiple sclerosis and rheumatoid arthritis [41]. Doxycycline has also been found to be able to inhibit Dengue virus replication in cell culture and is hypothesized to interact with the Dengue virus E protein to inhibit a conformational change which is required for virus entry into target cells [42]. More clinical studies will be needed in the future to determine whether treatment with doxycycline can provide significant therapeutic outcomes in DF, DHF and DSS patients by evaluating disease severity, survival rates and decreasing the required duration for hospitalization. Furthermore, it will be worthwhile to do small exploratory studies to determine whether doxycycline can provide any beneficial therapeutic effects to other virus-induced hemorrhagic fevers, although any therapeutic benefit with antibiotic treatment must be weighed against the potential risks associated with its use including the rise of antibiotic resistance. Interestingly, the use of antibiotics have been used as supportive antimicrobial therapy in Ebola patients in Guinea, but the direct effect of antibiotic treatment in this study was not known [33].

Zika virus is an important emerging mosquito-borne flavivirus that is causing large epidemics in the South America and Caribbean islands. In human infections, Zika fever is less severe compared with DHF and DF, but its pathogenesis is associated with the polyfunctional T-cell activation and involves the elevation of several serum cytokines [43]. In the acute infection phase, significant increases in the serum cytokines concentrations of IL-1b, IL-2, IL-4, IL-6, IL-9, IL-10, IL-13, IL-17, IFN-α-induced protein 10 (IP-10), RANTES and MIP-1α, compared with normal controls. In the late recovery phase, IL-10 levels were found to be highest [43]. Since both DF and Zika viruses are both mosquito-borne flaviviruses spread by the same vector, the *Aedes aegypti* mosquito, and share similar immunopathogenesis pattern in T-cell activation and inflammatory cytokine inductions, it is proposed by some that lessons learned from Dengue research may expedite the development of effective therapeutic and prophylactic strategies for Zika virus [44]

## Conclusion & future perspective

Many infectious disease and public health experts predict that zoonotic and mosquito-borne viruses will continue to emerge or re-emerge with increasing frequency in the future. Novel viruses will continue to evolve through naturally occuring mutations and pose a realistic threat to global public health as well as having significant impacts to the economy. Many of these emerging novel viruses are likely to be hard to protect against due to lack of approved antiviral drugs and vaccines that are available therefore preparedness through the identification of new/novel treatments and therapeutic strategies are essential for the future from a public health perspective. In particular, the development of approaches that target common events or pathways used by pathogenic viruses such as the induction of CS offer the potential for treatments that are effectice across different viral families and/or strains.

From each worldwide outbreak we are able to deepen the scientific understanding about how emerging viruses induce CS. Improving our knowledge with respect to the complex interplay between host's antiviral immune responses and the virus's ability to modify these responses and cause immune dysfunction will be critical for identifying new and effective therapies for treating the acute infections caused by many emerging viruses. It is envisaged that this increased understanding will lead to better treatment options to address the mortality and the morbidity caused by CS.

The effects of CS coupled with an acute viral infection in the host can be complex, overwhelming and potentially fatal to the host. Some of the promising therapeutic options for mitigating or treating CS highlighted in this review are listed in Figure 1A. It is likely that the most effective treatment strategy for virus-induced CS will be a comprehensive well-balanced strategy that is effective in downregulating inflammatory cytokines, reducing apoptosis and tissue damage/injury and ultimately restores the natural antiviral immune responses by maintaining physiological homeostasis in the body (Figure 1B). The use of a drug monotherapy may be insufficient to achieve this outcome. However, it is likely that the successful treatment of human patients infected with a CS-inducing emerging virus will need to encompass the use of a two- or three-pronged therapeutic strategy involving; an antiviral drug (if available), alongside the adjunct biological or chemical immune-modulating agent which suppresses CS and supportive therapy (fluid, electrolyte replacement and/or oxygen therapy).

**Table 1.** **Summary and selected examples of therapeutic option for cytokine storm induced by emerging viruses.**

**Treatment Strategy**	**Mode of action**	**Specific drug/product**	**Virus**	**Ref.**
Anti-inflammatory drugs	Direct anti-inflammatory effects resulting in, among others, reduction of inflammatory cytokines	NSAIDs	Pandemic influenza Ebola	[9] [38]

Biological or chemical modifiers	Suppression levels of inflammatory cytokines by affecting various signaling pathways	SIP1R agonists SERP-1 p38 and MAPK inhibitors	Human pandemic influenza A Ebola Ebola	[19,20] [34] [35]

Downregulation of inflammatory genes	Silencing of inflammatory master genes	DNAzyme DZ 13 for silencing of *c-Jun* gene	Avian influenza A/H5N1 virus	

Heat shock treatment	Induction of HSPs that play in a role in regulating innate & adaptive immunity	Short term heat shock (39°C for 4 h) to induction of HSP70	Avian influenza A/H5N1 virus	[29]

Combination therapy	Antiviral plus anti-inflammatory drugs	Zanamivir plus COX-2 inhibitors Oseltamivir plus S1P1R agonists	Avian and pandemic influenza	[17–19]

Supportive therapy	Fluid, oxygen and electrolytes	IV fluid resuscitation, crystalloids and oxygen therapy	Ebola	[33]

HSP: Heat shock protein; NSAID: Non-steroidal anti-inflammatory drug.

**Executive summary**

- Cytokine storm is an important underlying cause of mortality in patients infected with severe emerging viruses such as Ebola, Marburg, Middle Eastern Respiratory Syndrome coronavirus (MERS-CoV), pandemic influenza and others.
- There is currently no standard therapy to treat cytokine storm (CS) and there is no drugs which are approved specifically to treat CS.
- Clinical results for the reported use of corticosteroids and non-steroidal anti-inflammatory drugs as an adjunct therapy to antiviral drug to treat CS induced by pandemic influenza viruses had so far shown to be inconclusive, thus requiring more randomized controlled studies.
- There are a number of experimental treatment options which are currently in preclinical testing, and it is hoped that some of these leading drug candidates will be proven to be clinically safe and efficacious resulting in first drug to be specifically designed to treat cytokine storm.
- It is likely that treatment of these emerging virus infections will need to entail the use of antiviral drug in conjunction with an immune-modulating agent which suppresses CS and supportive therapy.
